# Prevalence and risk factors of louse- borne relapsing fever in high risk populations in Bahir Dar city Northwest, Ethiopia

**DOI:** 10.1186/1756-0500-7-615

**Published:** 2014-09-08

**Authors:** Mulat Yimer, Bayeh Abera, Wondemagegn Mulu, Belay Bezabih, Jemal Mohammed

**Affiliations:** Department of Microbiology, Immunology and Parasitology, College of Medicine and Health Science, Bahir Dar University, Bahir Dar, Ethiopia; Amhara National Regional State Health Bureau, Bahir Dar, Ethiopia; College of Health Science, Haramaya University, Haramaya, Ethiopia

**Keywords:** Ethiopia, Prevalence, Louse- borne relapsing fever

## Abstract

**Background:**

Louse- borne relapsing fever (LBRF) is a vector borne acute febrile illness caused by *Borrelia recurrentis* and the disease is more prevalent in the high risk groups like prisoners, yekoloremaries and street children. However, prevalence and risk factors of LBRF in these populations about the disease are not known. Therefore, the aim of this study was to determine the prevalence and risk factors of LBRF in high risk populations.

**Methods:**

A cross- sectional survey on prevalence and risk factors of LBRF in high risk populations in Bahir Dar city was conducted in December, 2012. For the study, blood was taken from the tip of the left ring finger of the participants by laboratory technicians and thick blood film was prepared from each participant and stained with 3% Giemsa for 30 min. The slides were examined and the result was reported as positive or negative using light microscopy and finally, data was also collected using a pre- tested questionnaire by face to face interviews.

**Results:**

Of the 407 study participants, 383 (94.1%) were males with the mean age of 31 years and 243 (59.7%) had no formal education. The prevalence of LBRF was 2.5% and the positivity rate of LBRF was highest in yekolotemaries (6.1%) followed by street children (4.9%). However, prisoners had nil and statistically significance association was observed between high risk populations and LBRF prevalence (p < 0.001). Those study participants who lived in mud houses had the highest positivity rate (2.2%), followed by those in wood houses (0.3%). However, those who lived in brick houses had nil. Study participants who had low levels of knowledge had the highest prevalence rate of LBRF.

**Conclusion:**

The overall prevalence of LBRF was 2.5% and the rate of positivity was highest in yekolotemaries, followed by street children. Therefore, health education should be given for these high risk populations.

## Background

Louse- borne relapsing fever (LBRF) is an acute febrile illness caused by *Borrelia recurrentis*, presenting with recurrence of characteristic febrile periods lasting for days alternating with afebrile periods [1]. The main manifestation is a recurring fever which coincides with massive numbers of bacteria in the blood. Its severity ranges from asymptomatic to fatal [2].

It is reported that 15 million cases of louse- borne relapsing fever (LBRF) and more than 5 million deaths occurred in Africa, Eastern Europe and Russia in the past [3]. It is now an important disease only in the North-Eastern Africa, specially the highlands of Ethiopia where an estimated 10,000 cases occur annually and affects mostly homeless people living crowded together in very unhygienic and crowded condition especially during rainy seasons [4]. Large outbreaks of LBRF have also occurred in Eritrea, Sudan, Somalia, and Ethiopia [5]. Transmission of spirochetes back to humans is accomplished when the louse is crushed while scratching and enter through the abraded skin [6, 7].

In Ethiopia, LBRF is within the top ten causes of hospital admissions, associated with significant morbidity and mortality [2]. For instances, in Southern Ethiopia (Hosanna hospital), LBRF admissions comprised 27% of total admissions [2] and 6% of mortality rate in South Western Ethiopia (Jimma hospital) [8]. Moreover, according to the Ethiopian health department report, it is the seventh most common cause of hospital admission and fifth most common cause of death [9].

Some of the risk factors are overcrowding like in military camps, prisons, street children sleeping areas, civilian population disrupted by war and other disasters [2, 6]. In spite of having the disease in these risk populations, yet there is paucity of information on prevalence and risk factors of the high risk populations about the disease in Ethiopia. Especially, it was not known in Bahir Dar. Therefore, the aim of this study was to determine the prevalence and risk factors of louse- borne relapsing fever in high risk populations.

## Methods

### Study area and period

A cross-sectional survey was conducted in December 2012 on prevalence and risk factors of louse- borne relapsing fever in high risk populations in Bahir Dar city.

Bahir Dar city is situated on the Southern shore of Lake Tana, the source of the Blue Nile (or Abay), in what was previously the Gojjam province. The city is located approximately 578 km North-West of Addis Ababa, having a latitude and longitude of 11°36′N 37°23′E11.6°N and an elevation of 1,840 meters above sea level. Based on the 2007 Census conducted by the Central Statistical Agency of Ethiopia, this city has a total population of 221,991, an increase of 130.90% over the population recorded in the 1994 census, of whom 108,456 are men and 113,535 women [10].

Study participants were high risk populations who were randomly selected and volunteered to participate in the survey. In this survey, the sample size was determined by considering that the prevalence of louse- borne relapsing fever in high risk populations was assumed to be 50%. Moreover, marginal error of 5% and 95% confidence interval (CI) with non response rates of 10% of the sample size was included. Using single proportion formula, 415 study participants were determined. The final study participants were sampled from each high risk populations, proportional to their number. Therefore, 234, 99 and 82 participants were included from prisoners, yekolotemaries and street children respectively.

However, 8 prisoners did not respond hence, survey questionnaire was completed in case of 407 participants to assess their knowledge and risk factors. Blood was taken from the tip of the left ring finger of the participants by laboratory technicians and thick blood film was prepared from each participant and stained with 3% Giemsa for 30 min. The slides were examined under 100 X objective using oil immersion. Finally, the result was reported as positive or negative using light microscopy to determine the prevalence of louse-borne relapsing fever [2].

### Data analysis

The quantitative data was checked for completeness, coded and fed in to SPSS version 16 and descriptive statistics (frequency, percentage, mean and standard deviation) were used primarily to summarize and describe the data to make it more graspable. For analytical statistic, *p* < 0.05 was considered statistically significant for association between variables.

A standardized questionnaire was developed and designed to address socio-demographic characteristics and risk factors of louse- borne relapsing fever and the following operational definitions were used.

#### Yekolotemaries

Are students who are from rural family of Ethiopia dedicated to learn in the Ethiopian Orthodox Church to become priests. They are grouped in low socio economic status; their livelihood is based on lobbing of foods like Ethiopian local foods from house to house. They live in mud houses and their hygienic condition is poor because they believe that being hygienic could reduce their learning capacities.

#### High risk populations

Are people who live in over-crowded high risk sites like prisons, military camps, yekolotemaries learning areas, street children sleeping areas etc. and are susceptible to the disease.

#### Knowledge

The knowledge that the respondent have regarding the prevention of louse- borne relapsing fever.

#### High levels of knowledge

Study participants who had 7–9 scores out of 9 questions about prevention of LBRF.

#### Moderate level

Study participants who had 4–6 scores out of 9 questions about prevention of LBRF.

#### Low level

Study participants who had 0–3 scores out of 9 questions about prevention of LBRF.

### Ethical consideration

Ethical clearance was obtained from Bahir Dar University, College of Medicine and Health Sciences and Regional health Bureau. The study participants were informed about the study in their language, including the purpose of the study. For those who were illiterate, the informed consent was read. Only those study participants who agreed and signed the informed consent were included. Finally, individuals’ positive for *Borrelia recurrentis* were treated accordingly with appropriate drugs.

## Results

0f the 407 study participants, majority of the participants were male 383 (94.1%). Male to female ratio were 15:1 and the mean age of the participants was 31 years with a standard deviation of 15.5. Regards to educational levels 243 (59.7%) had no formal education (Table 1).

**Table 1:** **Distribution of high risk populations and socio demographic characteristics on prevalence of louse- borne relapsing fever in Bahir Dar city, December, 2012**

	High risk populations			Total
	Yekolotemaries	Street children	Prisoners	
	(n = 99)	(n = 82)	(n = 226)	(n = 407)
Characteristic	No (%)	No (%)	No (%)	No (%)
**Sex**				
Male	99 (100)	58 (70.7)	226 (100)	383 (94.1)
Female	0	24 (29.3)	0	24 (5.9)
**Total**	**99 (24.3)**	**82 (20.1)**	**226 (55.5)**	**407 (100.0)**
**Age groups in years**				
11–20	79 (79.9)	27 (32.9)	33 (14.6)	139 (43.2)
21–30	18 (18.2)	16 (19.5)	79 (35)	113 (27.8)
31–40	0	13 (15.9)	50 (22.1)	63 (15.6)
41–50	2 (2)	5 (6.1)	31 (13.7)	38 (9.3)
≥ 51	0	21 (25.6)	33 (14.6)	54 (13.3)
**Total**	**99 (24.3)**	**82 (20.1)**	**226 (55.5)**	**407 (100.0)**
**Educational status**				
No formal	95 (96)	53 (64.6)	95 (42)	243 (59.7)
1–8 grades	4 (4)	25 (30.5)	74 (32.7)	103 (25.5)
9–12 grades	0	2 (2.4)	41 (18.1)	43 (10.6)
*Diploma &above	0	2 (2.4)	16 (7.1)	18 (4.4)
**Total**	**99 (24.3)**	**82 (20.1)**	**226 (55.5)**	**407 (100)**
**Marital status**				
Married	5 (5.1)	7 (8.5)	118 (52.2)	130 (31.9)
Single	93 (94)	44 (53.6)	82 (36.3)	219 (53.9)
Divorced	1 (1)	29 (35.4)	26 (11.5)	56 (13.8)
Widowed	0	2 (2.4)	0	2 (0.5)
**Total**	**99 (24.3)**	**82 (20.1)**	**226 (55.5)**	**407 (100)**

Note: n = Total numbers of types of study participants ; Diploma &above = Those who were diploma graduates and above.

The prevalence of louse- borne relapsing fever was 2.5% and the positivity rate of LBRF was highest in yekolotemaries (6.1%), followed by street children (4.9%) respectively. However, all of the prisoners had nil and statistically significance association was observed between high risk populations and LBRF prevalence (*p* < 0.001) (Table 2).

**Table 2:** **Distribution of prevalence of louse- borne relapsing fever in high risk populations in Bahir Dar city, December, 2012**

	High risk populations				
	Yekolotemaries	Street children	prisoners	Total	***p***value
*LBRF	(n = 99)	(n = 82)	(n = 99)	(n = 407)	< 0.001
**prevalence**	No (%)	No (%)	No (%)	No (%)	
Positive	6 (6.1)	4 (4.9)	0	10 (2.5)	
Negative	93 (94)	78 (95)	226 (100)	397 (97.5)	
**Total**	**99 (24.3)**	**82 (20.1)**	**226 (55.5)**	**407 (100)**	

Note: *LBRF – louse- borne relapsing fever, n = Total numbers of types of study participants.

**Figure 1:**
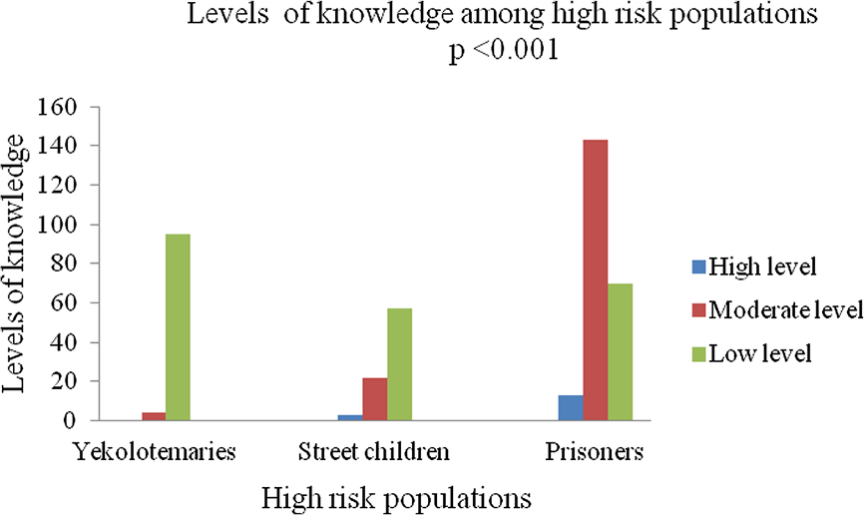
**Levels of knowledge among high risk populations in Bahir Dar city, December, 2012.**

Of the study participants, 219 (53.8%) had no information regarding LBRF, while only 35 (8.6%) had received some sorts of information from television and radio. Among the high risk populations, more than half of the prisoners heard some sort of information. However, none of the yekolotemaries had received information (Table 3).Levels of knowledge among high risk population revealed that yekolotemaries had no high levels of knowledge. However, most of the prisoners had moderate levels of knowledge and statistical significant association was observed (p < 0.001) (Figure 1).

**Table 3:** **Distribution of the sources which the high risk populations received information regards to louse- borne relapsing fever, in Bahir Dar city, December, 2012**

	High risk populations				
	Yekolotemaries	Street children	Prisoners	Total	
	(n = 99)	(n = 82)	(n = 226)	(n = 407)	
**Sources**	No (%)	No (%)	No (%)	No (%)	*p* value
*Tv and radio	0	0	35 (100)	35 (8.6)	
Friends	0	30 (26.3)	84 (73.7)	114 (28)	< 0.001
All of the above	0	2 (5)	37 (94.9)	39 (9.6)	
None of the above	99 (45)	50 (22.8)	70 (32)	219 (53.8)	
**Total**	**99 (24.3)**	**82 (20.1)**	**226 (55.5)**	**407 (100)**	

Note: *Tv: television, n = Total numbers of types of study participants.

Association between prevalence of LBRF and socio- demographic characteristics showed that males had higher positivity rate (1.7%) than females (0.7%) and statistically significance association was observed between sex and LBRF prevalence (*p* < 0.001). Regards to age, those groups from 11–20 showed the highest positivity rate (1.2%) and no statistically significant association was observed (*p* > 0.05). Educational status of high risk populations depicted that those groups who did not have formal education had highest positivity rate (2%). On the other hand, those who had grades from 9–12 and those who were diploma graduates and above, the positivity rate was nil. However, no statistically significant association was observed (*p* > 0.05) (Table 4).

**Table 4:** **Association between prevalence of louse- borne relapsing fever and socio-demographic characteristics in high risk populations in Bahir Dar city, December, 2012**

	Prevalence of louse- borne relapsing fever			
Characteristic	Positive	Negative	Total	***p***value
	No (%)	No (%)	No (%)	
**Sex**				
Male	7 (1.7)	376 (92.4)	383 (94.1)	*NA
Female	3 (0.7)	21 (5.2)	24 (5.9)	
**Total**	**10 (2.5)**	**397 (97.5)**	**407 (100.0)**	
**Age in years**				
11–20	5 (1.2)	134 (32.9)	139 (34.2)	< 0.490
21–30	2 (0.5)	111 (27.3)	113 (27.8)	
31–40	1 (0.3)	62 (15.2)	64 (15.7)	
41–50	1 (0.3)	37 (9)	38 (0.4)	
≥ 51	1 (0.3)	53 (13)	54 (13.2)	
**Total**	**10 (2.5)**	**397 (97.5)**	**407 (100.0)**	
**Educational status**				
No formal	8 (2)	235 (57.7)	243 (59.7)	< 0.188
1–8 grades	2 (0.5)	101 (24.8)	103 (25.3)	
9–12 grades	0	43 (10.6)	43 (11.8)	
*Diploma & above	0	18 (4.4)	18 (4.4)	
**Total**	**10 (2.5)**	397 (97.5)	**407 (100.0)**	

*NA: Not applicable; Diploma & above = Those who were diploma graduates and above.

**Figure 2:**
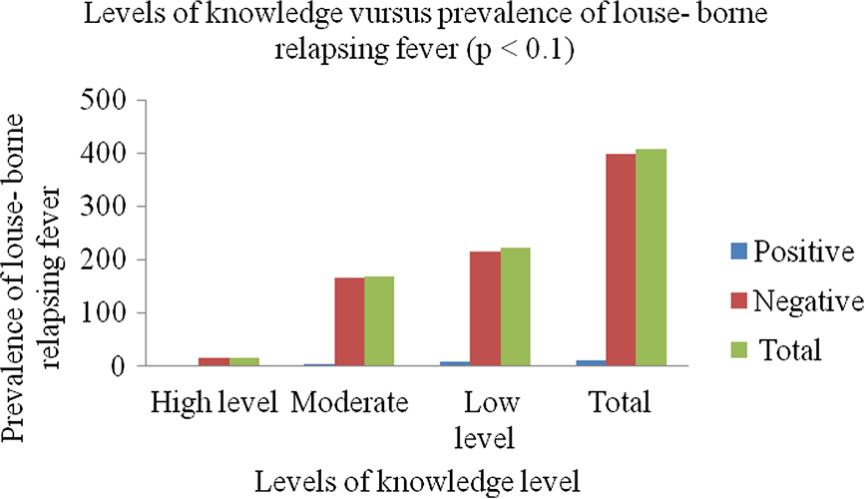
**Association between levels of knowledge and prevalence of louse- borne relapsing fever in high risk populations in Bahir Dar city, December, 2012.**

Association between prevalence of LBRF and social factors of high risk populations depicted that those study participants who washed their body and clothes once in two weeks had the highest positivity rate (1.7%). However, the least positivity rate (0.3%) was observed in those who washed twice or more a week and statistical significance association was seen (*p* <0.026). An attempt was made to determine the association of the type house in which the study participants live and prevalence of LBRF. This showed that those who lived in mud houses had the highest positivity rate (2.2%), followed by wood houses (0.3%). However, those who live in houses made up of bricks were not found with LBRF (Table 5). However, no statistical significant association was observed (*p* > 0.05).The association between knowledge and prevalence of LBRF revealed that those study participants who had low levels of knowledge had the highest prevalence of LBRF. However, prevalence of LBRF was nil in those who had high levels of knowledge (Figure 2).

**Table 5:** **Other factors associated with prevalence of louse- borne relapsing fever in high risk populations in Bahir Dar city, December, 2012**

	Prevalence of louse- borne relapsing fever			
	Positive	Negative	Total	***p***value
Characteristic	No (%)	No (%)	No (%)	
**Frequency of washing**				
Once in two weeks	7 (1.7)	107 (26.3)	114 (28)	< 0.026
Once a week	2 (0.5)	108 (26.5)	110 (27)	
Twice or more a week	1 (0.3)	182 (44.7)	183 (45)	
**Total**	**10 (2.5)**	**397 (97.5)**	**407 (100.0)**	
**Type of house**				
Mud houses	9 (2.2)	336 (82.6)	345 (84.8)	<0.394
Wood houses	1 (0.3)	17 (4.2)	18 (4.4)	
Bricks	0	44 (10.8)	44 (10.8)	
**Total**	**10 (2.5)**	**397 (97.5)**	**407 (100.0)**

## Discussion

In this study, the overall prevalence of LBRF was 2.5% and the positivity rate was highest in yekolotemaries (6.1%) (*p* < 0.001). This difference might be related to low levels of knowledge of LBRF among yekolotemaries as compared to street children and prisoners (Figure 1). Moreover, they did not have information regarding LBRF (Table 3). No positive result was observed in prisoners. This might be due to the fact that these study participants had high levels of knowledge about the disease (Figure 2).

In this study, we could not compare gender against the prevalence of LBRF due to absence of females in two high risk populations (yekolotemaries and prisoners) (Table 1). Regards to age, those groups from 11–20 yr showed highest positivity rate and almost all participants in these age groups were yekolotemaries (Table 1) and these groups had low levels of knowledge about the disease (Figure 2). The highest positivity rate was also seen in those study participants who had no formal education (Table 4) and did not know about the disease.

An attempt to determine the association between prevalence of LBRF and social factors of high risk populations revealed that those study participants who washed their body and clothes once in two weeks had the highest positivity rate (1.7%). It is clear that poor personal hygiene favours a higher prevalence of vectors for the diseases and occurs only when clothes are not changed or washed regularly [11]. Therefore, personal hygiene of study participants had significant association with prevalence of LBRF (*p* < 0.026) (Table 5).

Since this study was done in high risk populations hence, this might not represent the whole population. Moreover, there was no previous study done before on prevalence and risk factors of LBRF in high risk populations in Ethiopia and this is the first report.

## Conclusion

The overall prevalence of LBRF was 2.5% and the positivity rate of LBRF was highest in yekolotemaries, followed by street children. In this study, types of study participants and washing habits of the participant’s body and clothes were associated with prevalence of LBRF. Hence, types of study participants and washing habits of the participant’s body and clothes were an important factor in prevention of LBRF.

## Authors’ information

MY is an Assistant professor at College of Medicine and Health Sciences, Bahir Dar University in Medical Parasitology and head of Medical Parasitology.BA is an associate professor at College of Medicine and Health Science, Bahir Dar University in Medical Microbiology and department head of Microbiology, Immunology and Parasitology . WM is an Assistant professor at College of Medicine and Health Science, Bahir Dar University in Medical Microbiology. BB is field epidemiologist at Amhara National Regional State health Bureau and JM is lecturer at College of Medicine and Health Science, Haramaya University in Medical Parasitology.
